# Application of Ultrasound as Clean Technology for Extraction of Specialized Metabolites From Stinging Nettle (*Urtica dioica* L.)

**DOI:** 10.3389/fnut.2022.870923

**Published:** 2022-05-20

**Authors:** Jana Šic Žlabur, Sanja Radman, Nevena Opačić, Anamaria Rašić, Mia Dujmović, Mladen Brnčić, Francisco J. Barba, Juan Manuel Castagnini, Sandra Voća

**Affiliations:** ^1^University of Zagreb Faculty of Agriculture, Zagberb, Croatia; ^2^University of Zagreb Faculty of Food Technology and Biotechnology, Zagreb, Croatia; ^3^Food Science, Toxicology and Forensic Medicine Department, Faculty of Pharmacy, Preventive Medicine and Public Health, Nutrition and Food Science Area, Universitat de València, Vicent Andrés Estellés, València, Spain; ^4^Department of Agricultural Technology, Storage and Transport, University of Zagreb Faculty of Agriculture, Zagreb, Croatia

**Keywords:** ultrasound-assisted extraction, ethanolic extracts, polyphenols, ascorbic acid, pigments, antioxidant capacity

## Abstract

Nettle is a highly valued medicinal plant that is still largely neglected, both in terms of nutrition and use for pharmacological purposes. Tinctures, i.e., alcoholic extracts, are becoming increasingly popular nettle products, mainly because they allow better availability of phytochemicals and their stability over a longer period of time. The production of alcoholic extracts is a chemically demanding process that is still usually carried out using conventional techniques, which have numerous drawbacks. The use of green technologies such as ultrasound-assisted extraction (UAE), which is characterized by high efficiency of phytochemical extraction, shorter treatment time, and a much lower environmental footprint, is a suitable and sustainable solution. Therefore, the aim of this study is to determine the influence of the extraction method, conventional and ultrasound (by varying two ultrasound equipment systems), time and ethanol concentration on the extraction of specialized metabolites from nettle powder. Ultrasonic extraction using a probe system significantly contributed to increase the ascorbic acid yield, polyphenolic compounds, and antioxidant capacity of nettle extracts compared to conventional extraction. In addition, when a probe system was used during UAE, significantly less time was required for isolation of individual specialized metabolites compared to ultrasonic extraction in the bath. Ethanol concentration (50 and 80% v/v) also proved to be an important factor in the efficiency of extraction of specialized metabolites, with 80% ethanol being more effective for the isolation of ascorbic acid and pigment compounds (chlorophyll and carotenoids), while 50% v/v for the extraction of polyphenolic compounds. It can be concluded that extraction with the ultrasonic probe system is much more efficient in obtaining higher yields of specialized metabolites from nettle powder in a shorter time (average process duration 5–10 min) both compared to UAE in the bath and classical extraction. However, optimization of the key factors of time, solvent type, and ultrasonic power is necessary to maintain the nutritional quality of the nettle extract in order to obtain a final product with a high specialized metabolites content, antioxidant capacity, and functional value. The future application of alcoholic nettle extracts is based on the fact that these products have significant potential as functional foods and pharmacological preparations for the treatment of a number of but also to strengthen the immune system, mainly due to the rich nutritional composition and high content of various specialized metabolites. The prepared extracts can be safely taken orally by diluting the tinctures with water immediately before ingestion.

## Introduction

Stinging nettle (*Urtica dioica* L.) has been used for centuries as an important medicinal and food plant (1, 2). Although it is widespread and grows wild all over the world, its potential, especially in terms of its pharmacological and health properties, has not been fully exploited. This valuable medicinal plant is still considered a weed and is very often cleared and removed from farmlands, and frequently its natural habitats are increasingly threatened. The peculiarity of this species is reflected in its characteristic leaf structure, which is covered by stinging hairs, and which is ultimately the main obstacle to its consumption and processing, especially when fresh, as a vegetable (1, 3–5). Stinging nettle is known for its rich composition of various biologically active compounds, specialized metabolites (SM) and its complex chemical composition, which is why it has many beneficial effects on human health but is still the subject of numerous studies. It is interesting to note that all parts of the plant (root, stalk, leaves, and seeds) have a significant nutrient composition and a different SM profile. Depending on the harvest time in the year, the intended use of the nettle is determined. For medicinal purposes, the leaves (*Urticae folium*) are mostly used while also the whole plant (*Urticae herba*) can be used for pharmacological purposes, being collected from spring to flowering. Nettle is specific in terms of retrovegetation, i.e., cut nettle stalks are renewed in summer, so that nettle leaves and stalks can be harvested several times a year, depending on the specific abiotic environmental factors under which nettle grows or is cultivated (6, 7). From medicinal, pharmacological, and nutritional point of view, the leaves of stinging nettle are the most important part of the plant, as they contain numerous different specialized plant compounds and SM are specific primarily for their numerous health benefits. SM of nettle leaves can be divided into several categories: Terpenoids as components of the essential oil, terpene diols, terpene diol glucosides, α-tocopherol, most of which are carvacrol (8, 9), pigment compounds, of which, in addition to chlorophylls and carotenoids, β-carotene, violaxanthin, xanthophylls, zeaxanthin, luteoxanthin and lutein epoxide (6, 10, 11), polyphenolic compounds of which are mostly flavonoids such as kaempferol, quercetin (12–15), essential amino acids, vitamins such as vitamins C, K, vitamin B-complex, tannins, minerals most of which are calcium, iron, selenium, zinc, magnesium, phosphorus, potassium, and sodium (10, 11, 16–18). In addition, it is important to emphasize that *U. dioica* is considered the only plant species that contain choline acetyl transferase, an acetylcholine-synthesizing enzyme (19). Since the main limiting factor in the consumption of fresh stinging nettle leaves is the incandescent bulbs that often cause a burning sensation, it is often necessary to heat-treat the leaves for consumption, which often cause loss of many nutritional properties. For this reason, the need for the production of various nettle preparations in which all the nutritional properties are preserved is increasingly being applied and demonstrated. Also, as an increasing number of people is turning to a healthy lifestyle, many natural products are becoming more sought and popular (20, 21). One example of such a product is a tincture, an alcoholic extract of a particular medicinal plant that has a stronger and more concentrated effect (22–24). Nettle tincture is rich in nutrients and numerous SMs and therefore exhibits significant antioxidant properties and beneficial effects on human health. Thus, the alcoholic extract of nettle inhibits the activity of various bacteria (antimicrobial activity) (25), is effective against Benign Prostatic Hyperplasia (BPH) and urinary tract infections (26, 27), possesses cytotoxic, antitumor, and antimetastatic effects on breast cancer (28), can relieve the symptoms of osteoarthritis, and has anti-inflammatory properties (29, 30). In addition, nettle extract is used as a source of natural green color (natural dye) or pigment of natural origin for coloring soaps and shampoos, cosmetics, various lotions, toothpastes, antibacterial mouthwashes, and edible fats (7). It is used for sparse and weak hair, prevents alopecia (31).

The preparation of tinctures, i.e., alcoholic extracts, is a chemically demanding process that is usually still carried out using conventional techniques based on the basic postulates of solid–liquid extraction. Conventional techniques often entail numerous disadvantages: prolonged extraction time, use of harmful organic solvents, use of higher temperatures, as well as negative effects on phytochemicals. Therefore, recently, various modern extraction methods have been increasingly developed and researched, primarily based on environmentally friendly technologies. The main advantage of such technologies is lower environmental impact, energy efficiency, significantly shorter process duration, and significant preservation of nutritional properties of the final product (extract), which is characterized by the higher concentration of valuable biologically active compounds and consequently increased antioxidant activity (32–34). One such non-invasive extraction technique, that is characterized as a clean and green technology, is ultrasound-assisted extraction (UAE). The use of UAE is based on the phenomenon of transient cavitation, a mechanism of sonication in the liquid medium (35). Once propagated through a liquid medium, ultrasound, with its properties below 20–100 kHz frequency and intensity of 5–300 W/cm^2^, enables high reproducibility in a shorter time, easier handling, lower temperatures during processing, and the use of smaller amounts of solvents (36, 37). Another significant advantage of sonication for extraction is the improved preservation of nutrients. In particular, numerous studies demonstrate the preservation of bioactive compounds during sonication, high reproducibility in a shorter time, easier handling, lower temperatures during processing, and the use of lower amounts of solvents (33, 34, 38, 39). To achieve higher yields of specialized metabolites from plant material during solid–liquid extraction using high-intensity ultrasound, several variables need to be optimized, generally classified as physical (the ultrasound waves applied during UAE and the equipment used), medium-dependent (solvent properties, temperature, and the presence of gasses), and matrix-dependent parameters (matrix, structure, particle size, and solid–liquid ratio) (40). There are three types of laboratory ultrasound equipments that are commercially available and most commonly used: the ultrasound bath, the ultrasound probe (or horn) system, and the cup-and-horn system. These devices differ primarily in the amount of power supplied to the system, which ultimately affects the cavitation process and the efficiency of the process itself. In general, probe and cup-horn systems are more efficient compared to baths, mainly because the amount of acoustic energy can be controlled by adjusting the amplitude and ultrasound intensity. Also, it is important to note that those devices are usually supplied with higher ultrasound power compared to the baths, which significantly reduces the time of extraction. Nevertheless, ultrasonic baths are still the most widely used and popular systems for ultrasonic application, mainly because of the much lower price, but also because they are suitable for sensitive materials due to the lower cavitation effect (41–43). Therefore, the aim of this study is to determine the influence of the extraction method, conventional and ultrasound (by varying two ultrasound equipment systems), time and ethanol concentration on the extraction of specialized metabolites from nettle powder.

## Materials and Methods

### Plant Material

The powder was prepared from fresh leaves of cultivated stinging nettle grown in a greenhouse at the University of Zagreb Faculty of Agriculture in the Department of Vegetable Cultivation. The nettle was grown in a greenhouse in a floating hydroponic system during the spring-summer growing season from March 18 (sowing) to June 8, 2021 (harvest of the above-ground mass). Nettle was placed in the pools filled with a nutrient solution containing a combination of salts suitable for growing leafy vegetables. The recommended values for the basic parameters of the nutrient solution were temperature 20 to 24°C, pH 5.8 to 6.2, dissolved oxygen content 4 to 9 mg/L, and EC value 2.5 to 3.2 dS/m. Nettles were repeatedly mowed during the pre-flowering period at a height of 15–20 cm above the lower two nodules. Fresh nettle leaves from the third mowing period in July 2021 were used to prepare the powder. Immediately after mowing, the above-ground part of the plant was taken to the laboratory of the Department of Agricultural Technology, Storage and Transport of the University of Zagreb Faculty of Agriculture where the nettle leaves were separated from the stems by hand and laid out to dry in natural conditions. The leaves were spread in one layer on cardboard paper and dried in a ventilated room at an average room temperature of 25°C and relative humidity of 73%. The nettle leaves were dried to average water content in the dry material of 10%. The final water content in the dry plant biomass was determined by drying at 105°C to constant mass using a standard laboratory method (44). The drying process under natural conditions to the desired water content took 5 days. The dried nettle leaves were ground to a powder using a laboratory mill (IKA MF-10, IKA®-Werke GmbH & Co., Staufen, Germany). The ground sample was passed through a system of several sieves with different pore sizes to determine an average powder particle size of 1 mm.

### Preparation of Alcoholic Extracts

Regardless of the extraction method, 2.0 g ± 0.01 nettle powder (Sartorius, Entris® II Essential, Zagreb, Croatia) was weighed into laboratory beakers with a volume of 300 ml. Ethanol with a volume of 130 ml was used as an organic solvent for the extraction. For the purpose of the experiment, i.e., preparation of nettle powder tincture, two different concentrations of ethanol were varied: 50% (v/v) and 80% (v/v). Part of the samples was separated for treatment by ultrasonic probe system, part for treatment in an ultrasonic bath, while classical extraction by solid–liquid technique was a control sample. The classical extraction was performed in such a way that the prepared samples were left at room temperature for 24 h with occasional stirring. After the designated extraction time, the samples were filtered through Whatman filter paper and used for further analysis. The setup of the experiment is shown in Table 1.

**Table 1:** Nettle powder extraction experiment plan.

**Extraction method**	**Solvent concentration (v/v)**	**Solvent volume (mL)**	**Time of extraction (min)**	**Output power (W)**	**Amplitude (%)**	**Sample ID**
Conventional solid-liquid	EtOH, 50%	130	1,440	-	-	SL-50
Conventional solid-liquid	EtOH, 80%	130	1,440	-	-	SL-80
UAE probe	EtOH, 50%	130	5	200	20	PS-50-5
UAE probe	EtOH, 50%	130	10	200	20	PS-50-10
UAE probe	EtOH, 50%	130	15	200	20	PS-50-15
UAE bath	EtOH, 50%	130	10	140	-	B-50-10
UAE bath	EtOH, 50%	130	15	140	-	B-50-15
UAE bath	EtOH, 50%	130	30	140	-	B-50-30
UAE probe	EtOH, 80%	130	5	200	20	PS-80-5
UAE probe	EtOH, 80%	130	10	200	20	PS-80-10
UAE probe	EtOH, 80%	130	15	200	20	PS-80-15
UAE bath	EtOH, 80%	130	10	140	-	B-80-10
UAE bath	EtOH, 80%	130	15	140	-	B-80-15
UAE bath	EtOH, 80%	130	30	140	-	B-80-30

### Ultrasonic-Assisted Extraction

**Figure 1:**
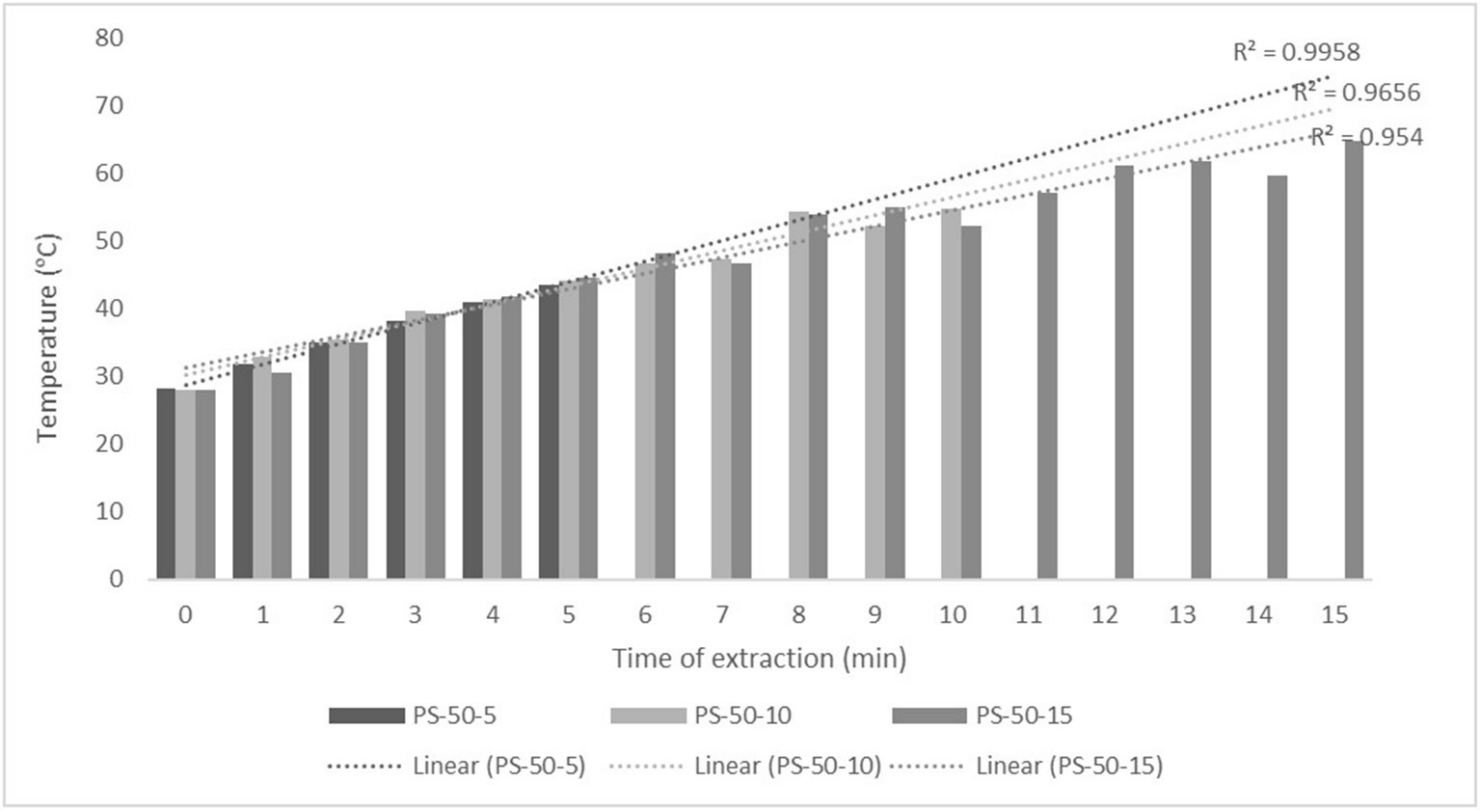
Temperature (°C) of nettle extracts with 50% v/v ethanol during the ultrasound-assisted extraction (UAE) by probe system for 5 min (PS-50-5), 10 min (PS-50-10), and 15 min (PS-50-15).

**Figure 2:**
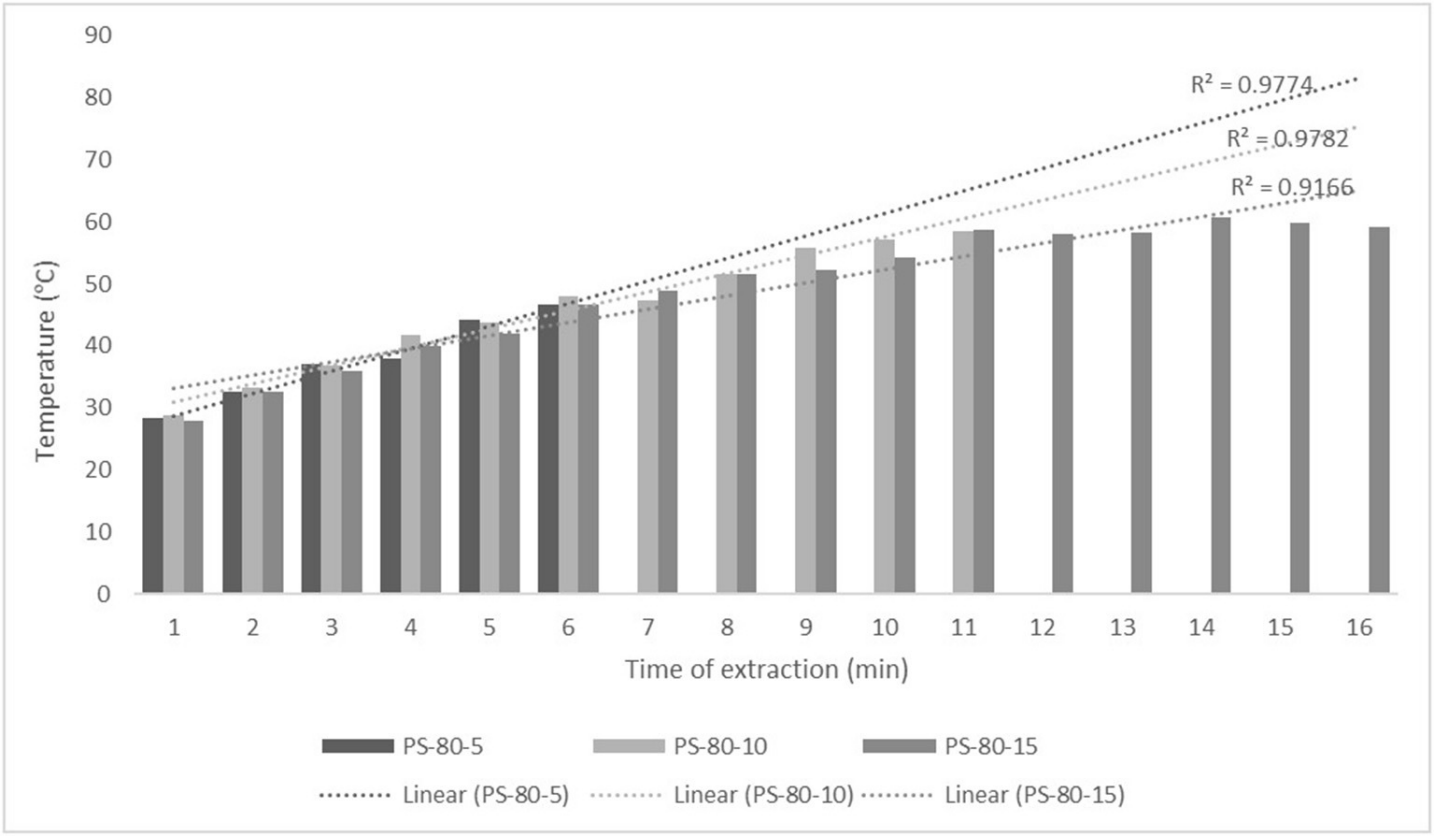
Temperature (°C) of nettle extracts with 80% v/v ethanol during the UAE by probe system for 5 min (PS-80-5), 10 min (PS-80-10), and 15 min (PS-80-15).

**Figure 3:**
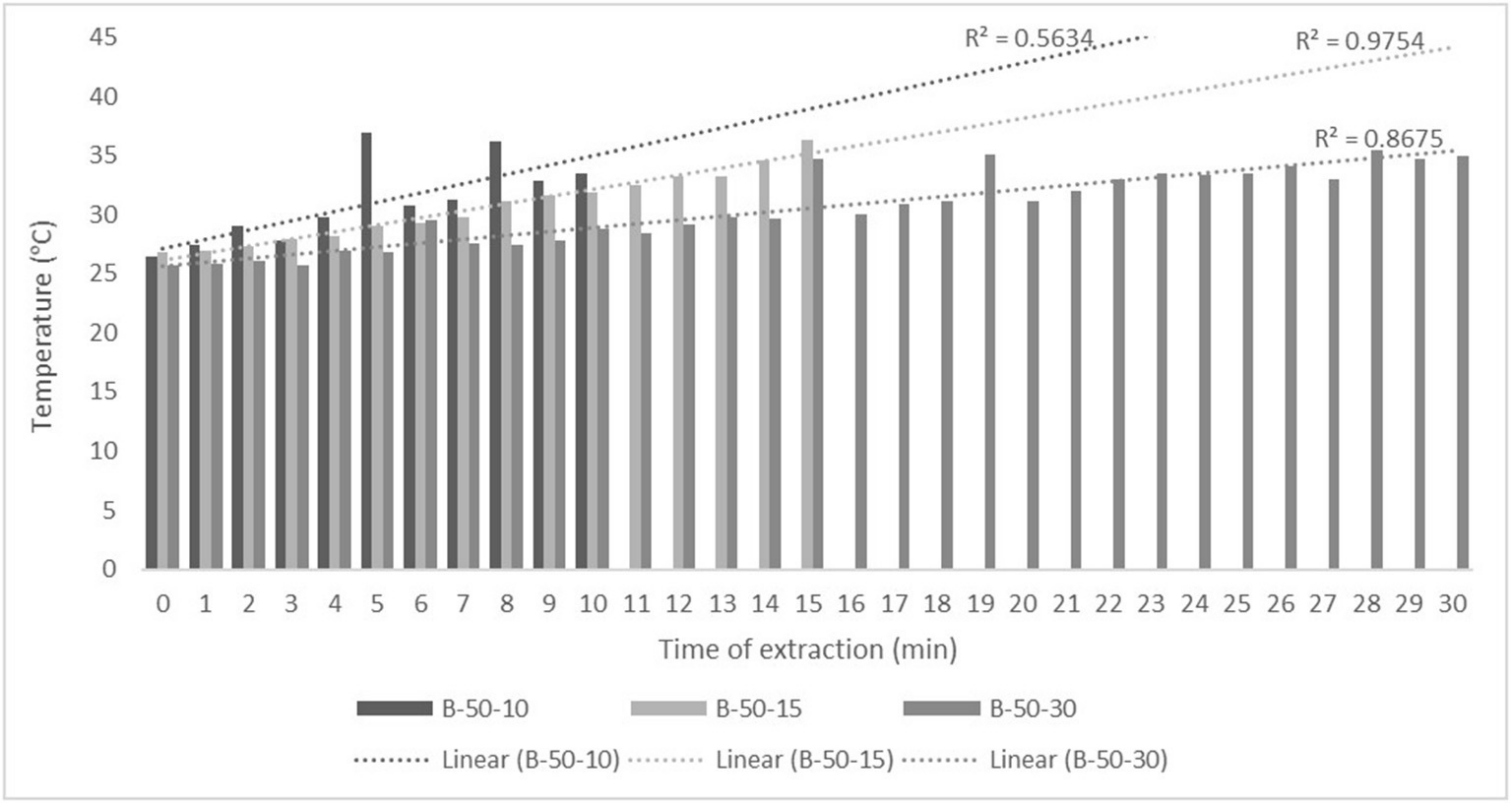
Temperature (°C) of nettle extracts with 50% v/v ethanol during the UAE in the ultrasonic bath for 10 min (B-50-10), 15 min (B-50-15), and 30 min (B-50-30).

**Figure 4:**
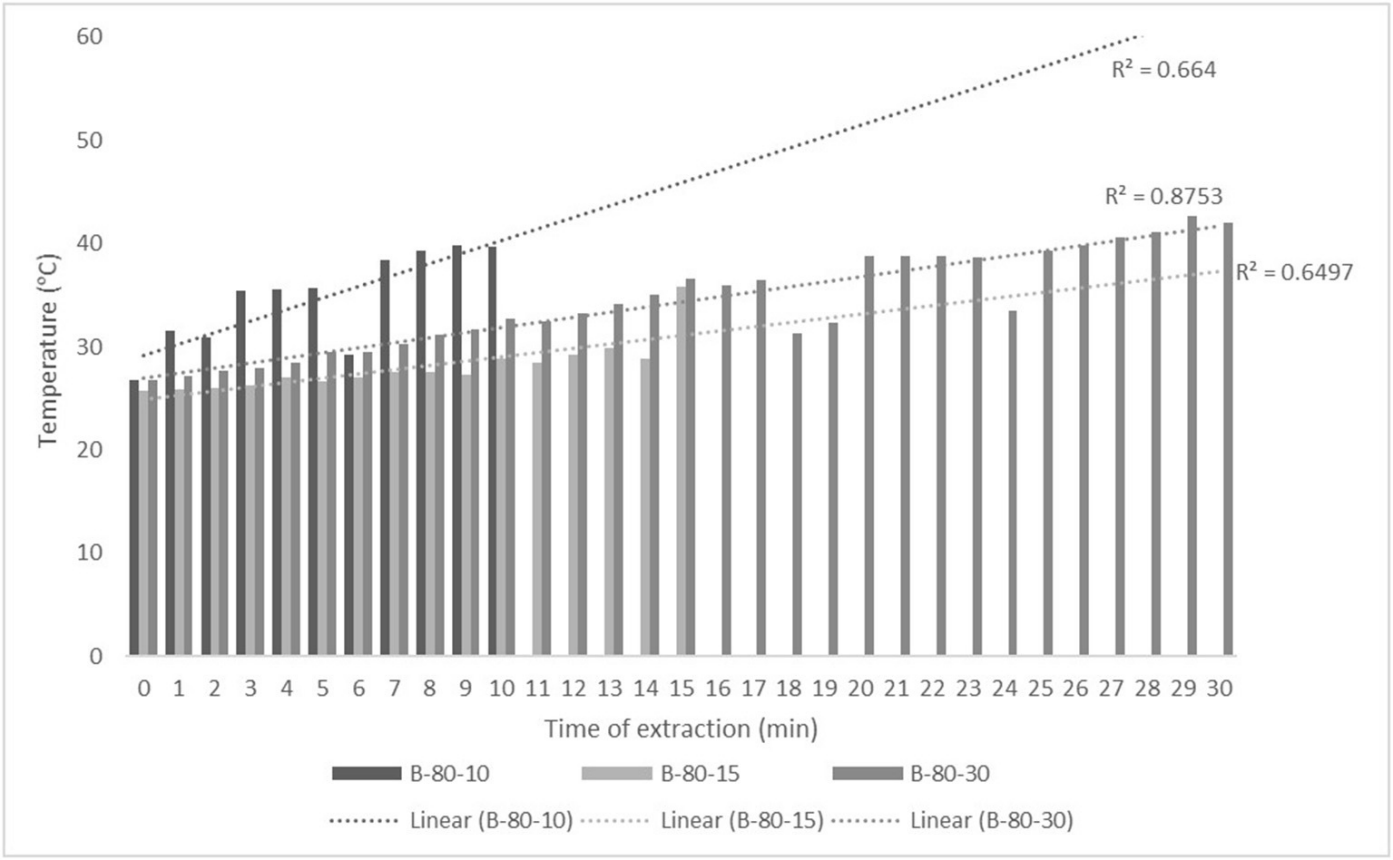
Temperature (°C) of nettle extracts with 80% v/v ethanol during the UAE in the ultrasonic bath for 10 min (B-80-10), 15 min (B-80-15), and 30 min (B-80-30).

For the purposes of UAE, the equipment type was varied. UAE treatment with an ultrasonic probe system (Bandelin HD 2000.2, Germany) was performed with a device with a nominal maximum power of 200 W (30% amplitude used) and a probe diameter of 13 mm inserting it directly into the prepared sample, with varying treatment times of 5, 10, and 15 min. The UAE treatment in the ultrasonic bath (Bandelin RK 103H, Germany) was carried out by placing the samples in beakers in an ultrasonic bath with a frequency of 35 kHz and a nominal maximum power of 140 W, with varying treatment times of 10, 15, and 30 min. Since the nominal output power of the devices used was not the same, different treatment times were adapted to ultrasound efficiency for each type of ultrasound device. After each treatment, samples were filtered through Whatman filter paper to separate the solid phase and obtain a liquid nettle extract. During sonication, the temperature of the samples was measured with a laser thermometer (Raytek–MiniTemp FS, Raytek, Toronto, ON, Canada) in time intervals of 60 s to monitor the temperature change during ultrasonic treatment, as shown in Figures 1–**4**.

### Determination of Specialized Metabolites Content of Nettle Alcoholic Extracts

The analysis of specialized metabolites included determination of the: (i) ascorbic acid (AsA) content (mg/100 g) by titration with 2,6-dichlorindophenol (DCPIP) according to the standard method (45). AsA was isolated from the nettle alcoholic extracts by homogenizing the 10 g ± 0.01 of the extract with a total of 100 ml of 2% (v/v) oxalic acid. The prepared solution was allowed to stand for about 20 min, filtered through Whatman filter paper and 10 ml of filtrate was used for titration with DCPIP. Titration with freshly prepared DCPIP was carried out until pink coloration appeared. The final AsA content was calculated according to Equation (1) and expressed as mg/100 g fresh weight (fw).


(1) $$\begin{array}{l} \text{AsA }\left(\text{mg}/100\text{g fw}\right)\;=\;\frac{\text{V }\times \text{ F}}{\text{D}}\;\times \;100 \end{array}$$

where V is the volume of DCPIP (ml); F is the factor of DCPIP; and D is the sample mass used for titration in 10 ml of extract; (ii) total phenol content (TPC) according to the method described by Shukla et al. (46) based on the appearance of blue coloration in the reaction with Folin–Ciocalteu reagent. The reaction procedure was as follows: 1 ml of the nettle alcoholic extract and 1 ml of the Folin–Ciocalteu reagent diluted with distilled water (1:2) were added in a volumetric flask with a volume of 50 ml and allowed to stand for 3 min. Additionally, 3 ml of a saturated sodium carbonate solution (Na_2_CO_3_) was added, the flask was filled to the mark with distilled water and allowed to stand for 3 h at room temperature with intermittent shaking. The absorbance of the blue color was measured spectrophotometrically (Shimadzu 1900i, Shimadzu Co., Kyoto, Japan) at 750 nm with distilled water as a blank; (iii) total flavonoids content (TFC) according to the Abou-Arab and Abou-Salem (47) as follows: 1 ml of the extract, 1 ml of 20% HCl (v/v), and 0.5 ml formaldehyde were added in a volumetric flask volume of 25 ml. The prepared samples were blown with nitrogen (N_2_) and allowed to stand for 24 h at room temperature, after which, the same Folin–Ciocalteu reaction as for the TPC was carried out. As a standard for TPC was used gallic acid, while for TFC catechin and the final content of TPC and TFC in the nettle alcoholic extracts were expressed as mg GAE/100 g fw for TPC and as mg CTH/100 g fw for TFC. Total non-flavonoid content (TNFC, mg GAE/100 g fw) was mathematically expressed as the difference between TPC and TFC.

### Determination of Pigment Compounds Content of Nettle Alcoholic Extracts

From the group of pigment compounds chlorophyll a (Chl_a), chlorophyll b (Chl_b), total chlorophylls (TCh), and total carotenoids (TCA) content were determined according to the method described by Holm (48) and Wettstein (49). For the extraction of pigments from nettle alcoholic extracts, 5 g ± 0.01 of the extract was weighed, and a total of 15 ml of acetone (p.a.) was added three times. After each addition of acetone, the samples were homogenized using a laboratory homogenizer (IKA, UltraTurrax T-18, Staufen city, Germany). The final solution was filtered through Whatman filter paper and transferred to a 25 ml volumetric flask filled with acetone to the mark. Absorbance was measured spectrophotometrically (Shimadzu UV 1900i, Duisburg Germany) at 662, 644, and 440 nm using acetone as a blank. Holm–Wettstein equations were used to quantify chlorophyll and carotenoid content according to Equation (2), while the final content was expressed in μg/g.


(2) $$\begin{array}{l} Chl\text{\_}a\;=\;9.784\;\times \;A_{662}\;-\;0.990\;\times \;A_{644}\left[mg/L\right]\\ Chl\text{\_}b\;=\;21.426\;\times \;A_{644}\;-\;4.65\;\times \;A_{662}\left[mg/L\right]\\ TCh\;=\;5.134\;\times \;A_{662}\;+\;20.436\;\times \;A_{644}\left[mg/L\right]\\ TCA\;=\;4.695\;\times \;A_{440}\;-\;0.268\;\times \;TCh\;\left[mg/L\right] \end{array}$$

### Determination of Antioxidant Capacity of Nettle Alcoholic Extracts

The antioxidant capacity was determined by performing the 2,20-azinobis (3-ethylbenzothiazoline-6-sulfonic acid) (ABTS) assay according to the method described by Re et al. (50). Trolox (6-hydroxy-2,5,7,8-tetramethylchroman-2-carboxylic acid, TE) was used as the antioxidant standard, while the standard Trolox solution (2.5 mM) was prepared in ethanol (80% v/v). For the preparation of the ABTS radical solution (ABTS+), 5 ml of ABTS solution (7 mM) and 88 ml of potassium persulphate solution (140 mM) were mixed and left in the dark at room temperature for 16 h. On the day of analysis, a 1% ABTS+ solution (in 96% ethanol) was prepared. A total of 160 μL of the nettle alcoholic extract was directly injected into the cuvette and mixed with 2 mL of 1% ABTS+ while absorbance was measured at 734 nm (Shimadzu 1900i, Duisburg, Germany). The final antioxidant capacity results were calculated from the calibration curve and expressed in μmol TE /L (according to Trolox). ABTS, potassium persulfate, and Trolox (6-hydroxy-2,5,7,8-tetramethylchroman-2-carboxylic acid) (TE) were purchased from Sigma-Aldrich (St. Louis, MO, USA).

### Determination of Physicochemical Parameters of Nettle Alcoholic Extracts

The physicochemical properties of the ethanolic stinging nettle extracts were determined within the research: (i) density (g/cm^3^) by digital densitometer (Densito 30PX, Mettler-Toledo, Switzerland); (ii) electrical conductivity (μS/cm) by conduct meter (Mettler Toledo, SevenEasy Conductivity, Switzerland); (iii) pH value using a digital pH-meter (Sevenmulti, Mettler Toledo, Switzerland); (iv) total acid content (TA, %) by potentiometric titration according to the AOAC (44) and (v) chromaticity parameters (L^*^, a^*^, b^*^, C, h°) were determined according to the CIELab method using a colorimeter (ColorTec PCM+, PCE Instruments, Southampton, UK).

### Statistical Analysis

All treatments were performed in triplicate, as in conventional and ultrasonic-assisted extraction. A generalized linear model was used that included replicate, extraction method: conventional, UAE (probe system, bath), ethanol concentration (50 and 80% v/v), and time as categorical predictors. For the analysis procedures, PROC GLM in the SAS software package, version 9.4, was used (51). The obtained data were analyzed with ANOVA. Means were compared using the *t*-test (LSD) and considered significantly different at *p* ≤ 0.0001. Different letters are indicated in the tables to denote significant differences between the means within each column, and the ±SD was also indicated. Parameters were then analyzed classified using principal components analysis (PCA, Minitab v.17). A regression analysis was performed between the color parameters (L, a^*^, b^*^, C, and h°) and the concentration of chlorophylls (a, b, and total) and carotenoids (Minitab v.17).

## Results and Discussion

### Specialized Metabolites Content of Nettle Alcoholic Extracts

The properties of the phytochemicals targeted for extraction (extracted compound), especially the structure of the molecule, sensitivity to process conditions such as temperature (thermolabile compounds), solubility as a function of solvent polarity, etc., are also very important factors that should be considered during UAE. Vitamins are among the nutrients that are very sensitive to process conditions, especially to higher temperature and longer extraction time. Therefore, optimization of UAE process variables, especially ultrasonic power (52, 53), to preserve labile biologically active compounds is a major challenge. The results of AsA content in the samples of alcoholic nettle extracts are shown in Table 2. As for the combined treatments, the highest AsA levels were observed in samples treated with UAE in a system with an ultrasonic probe, with an average AsA value of 72.88 mg/100 g. Comparing the AsA yields obtained in alcoholic nettle extracts by different extraction methods (conventional and UAE), regardless of solvent concentration, type of ultrasonic equipment, and time, it can be observed that significantly higher AsA values were recorded in the samples treated with UAE, on average more than 3.5 times higher values than in the classically treated samples. These results can be supported by other literature data, which also emphasize the efficiency of UAE in obtaining high AsA yields in different matrix extracts (54–56). The type of ultrasonic equipment and ethanol concentration (water content in ethyl alcohol) significantly affected the AsA content in nettle extracts. The ultrasonic probe system was revealed to be significantly more effective than the ultrasonic bath in AsA extraction. About 2-fold higher AsA content was observed in alcoholic nettle extracts treated with the ultrasonic probe system, regardless of the ethanol concentration used. Indeed, ultrasound power as a crucial parameter has a strong influence on the efficiency of UAE extraction, and in general, the use of high intensities (influenced by the amplitude setting) leads to higher extraction yields. The main reason for this effect is the generation of strong shear forces, which are achieved to a much greater extent when using the probe and cup-horn systems (probes) compared to the bath system. In general, the higher power delivered (acoustic energy → cavitation energy) is the main advantage of using an ultrasonic probe system in the application of UAE processes compared to ultrasonic baths, where the main disadvantage is the lower power (41, 42). In the combination of the equipment used in this study, the probe system (nominal output power 200 W) delivered higher ultrasonic energy when compared to the ultrasonic bath (output power 140 W), which is the main reason for the more effective extraction of AsA with the ultrasonic probe. However, it is important to emphasize that high intensities or the application of higher ultrasound power without optimization and control can have opposite effects. They can lead to overheating of the system, resulting in degradation of thermolabile compounds (such as vitamins), evaporation of solvents, generation of liquid agitation, loss of ultrasound waves, and reduction of transient cavitation efficiency (43, 57, 58). As for the temperature of the medium measured during the UAE application, it can be observed, both in the probe system (Figures 1, 2) and in the bath (Figures 3, 4), that the application of a higher ultrasound power delivered by the treatment in the probe system did not cause overheating of the matrix, i.e., the medium, and thus did not cause degradation of the AsA content. Moreover, it is worth mentioning that the application of higher ultrasonic power significantly shortens the extraction time, which is another valuable advantage of using ultrasonic probe systems. A longer extraction time can cause undesirable changes and also lead to the degradation of the extracted compounds (56, 59). According to the results of this study, for example, significantly higher AsA yields were recorded during the UAE treatment with the probe system for a duration of 15 min than for the same treatment duration in the ultrasonic bath, regardless of the ethanol concentration. When using 80% ethanol (v/v), the influence of processing time by UAE is even more pronounced. In this case, only 5 min of treatment in the ultrasonic probe system was sufficient to obtain significantly higher AsA values than a half-hour treatment in the bath with the same ethanol concentration used. Optimizing the processing time minimizes not only the retention of bioactive compounds but also the energy consumption, thus increasing the efficiency of UAE extraction. In order to successfully perform the extraction and obtain an adequate yield of the extracted compound, another important parameter is also the type of solvent and its properties, such as polarity, viscosity, density, etc. The acoustic power as a direct result of the ultrasonic processor depends on the type of solvent, mainly due to the loading of the acoustic probe, which depends on the solvent “resistance” or acoustic impedance. In general, water has a higher acoustic impedance than ethyl alcohol (41, 60), so the acoustic/transient energy generated by the ultrasonic processor is better distributed in solvents with a greater percentage of ethyl alcohol. These phenomena can be confirmed by the results of this study by the significance of the factor interactions (Table 2), respectively, with the highest observed significance of the interactions (*p* ≤ 0.0001) for the combination, i.e., the interaction of extraction method and solvent type (EM × S). As mentioned earlier, it is important to emphasize that vitamins (including vitamin C) are present in various matrix and food systems as chemically bound complexes, which have lower digestion and absorption efficiency compared to the free forms of vitamins (61). In this context, ultrasound energy stimulates bond breaking between vitamins and their coenzymes, thus affecting the higher bioavailability of each vitamin (53).

**Table 2:** Specialized metabolites content of nettle alcoholic extracts.

**Sample ID**	**AsA** **(mg/100 g)**	**TPC** **(mg GAE/100 g)**	**TNFC** **(mg GAE/100 g)**	**TFC** **(mg CTH/100 g)**	**Ant_cap** **(μmol TE/L)**
Classic extraction					
SL-50	6.22^g^ ± 0.77	371.97^k^ ± 0.33	223.91^h^ ± 0.41	148.07^i^ ± 0.47	2184.93^h^ ± 4.99
SL-80	10.73^fg^ ± 0.38	331.74^i^ ± 0.22	145.29^g^ ± 1.28	186.66g ± 1.49	2511.93^a^ ± 0.79
Ultrasonic-assisted extraction					
PS-50-5	10.12^fg^ ± 0.36	570.41^e^ ± 1.59	270.91^f^ ± 1.48	300.17^c^ ± 3.87	2385.09^f^ ± 0.99
PS-50-10	20.79^def^ ± 0.74	672.02^b^ ± 0.49	306.70^bc^ ± 1.39	365.32^a^ ± 1.14	2247.5^g^ ± 0.36
PS-50-15	41.49^bc^ ± 0.64	682.82^a^ ± 1.51	323.34^a^ ± 1.31	359.48^a^ ± 1.22	2455.87^cd^ ± 3.61
B-50-10	29.88^cd^ ± 3.61	467.98^g^ ± 0.59	302.74^c^ ± 1.85	165.25^h^ ± 2.19	2372.79^f^ ± 0.74
B-50-15	30.44^bcd^ ± 3.31	462.99^h^ ± 0.15	279.60^e^ ± 3.47	183.39^g^ ± 5.32	2406.29^ef^ ± 0.55
B-50-30	27.14^de^ ± 3.53	500.81^f^ ± 0.79	289.33^d^ ± 4.10	211.48^f^ ± 4.86	2376.87^f^ ± 3.05
PS-80-5	43.59^b^ ± 3.73	416.45^j^ ± 1.41	186.05^i^ ± 0.58	230.39^e^ ± 1.97	2519.48^a^ ± 1.79
PS-80-10	68.69^a^ ± 3.76	576.66^d^ ± 0.73	291.26^d^ ± 1.17	285.40^d^ ± 1.51	2505.72^ab^ ± 1.35
PS-80-15	77.07^a^ ± 3.53	619.02^c^ ± 0.59	311.04^b^ ± 0.34	307.98^b^ ± 0.92	2472.27^bc^ ± 0.59
B-80-10	14.32^efg^ ± 1.52	141.60^l^ ± 0.19	86.28^j^ ± 0.61	55.33^j^ ± 0.76	2472.27^bc^ ± 1.06
B-80-15	10.79^fg^ ± 0.68	104.11^n^ ± 0.57	77.93^k^ ± 0.90	26.19^k^ ± 1.43	1796.75^i^ ± 3.28
B-80-30	8.83^fg^ ± 0.52	136.89^m^ ± 3.8	86.82^j^ ± 0.43	50.08^j^ ± 3.46	2429.65^de^ ± 2.59
ANOVA	*p* ≤ 0.0001	*p* ≤ 0.0001	*p* ≤ 0.0001	*p* ≤ 0.0001	*p* ≤ 0.0001
LSD	13.49	2.9911	6.3106	7.3311	
EM × S	0.0001	0.0001	0.0001	0.0001	0.0050
EM × T	0.2466	0.6653	0.5959	0.7745	0.0036
S × T	0.2600	0.2700	0.1113	0.5066	0.0014
EM × S × T	0.0001	0.0001	0.0001	0.0001	0.0001

*AsA, ascorbic acid content; TPC, total phenol content; TNFC, total non-flavonoid content; TFC, total flavonoid content; EM × S, interaction of the extraction method and solvent concentration; EM × T, interthe action of extraction method and time; S × T, interthe action of solvent concentration and time; EM × S × T, the interaction of extraction method, solvent concentration and time. Results are expressed as mean ± standard deviation. Different letters indicate significant differences between mean values*.

Besides strong UAE efficiency on obtaining high AsA yield in nettle alcoholic extracts, the efficiency of UAE on polyphenolic compounds (total phenols, flavonoids, and non-flavonoids) was also observed within this study (Table 2). On average about 27% higher content of total phenolics (TPC), 28% higher content of total flavonoids (TFC), and 27% higher content of total non-flavonoids (TNFC) were recorded in UAE treated samples, regardless of the equipment type, solvent concentration, and time of extraction, compared to the classically treated ones. Obtained results of significantly higher content of polyphenolic compounds in nettle alcoholic extracts treated by UAE can be supported with other literature data which also emphasize the positive effect of ultrasound on polyphenolics (62–64). Again, as for the AsA content, in relation to varied ultrasound equipment types, probe system was more effective in the extraction of analyzed polyphenols (TPC, TFC, and TNFC). In extraction with 50% ethanol by ultrasonic probe system, regardless of the varied time, even about 35% higher TPC, 83% higher TFC, and about 4% higher TNFC content was determined compared to the extraction in the ultrasonic bath, while in the UAE extraction with 80% ethanol by ultrasonic probe system TPC was about 13% higher, TFC even 6 times and TNFC about 3 times higher compared to the extraction in bath. The obtained results also support that the greatest efficiency of the UAE extraction of polyphenolic compounds is under the main influence of ultrasonic power, whereby higher amounts of ultrasound power delivered and distributed in liquid media will increase the effect of cavitation bubbles collapse, respectively promote transient cavitation phenomena. Namely, collapsing of cavitation bubbles generates shockwaves and accelerates inter-particle collision causing the fragmentation and even degradation of cellular structure. The fragmentation of cellular structure cause decrease in particle size, increase surface area, and high mass transfer rates in the boundary layer of solid matrix thus providing better solubilization of the cell components, first of all phytonutrients such as bioactive compounds (62, 63). Higher amounts of ultrasound power promote significant shortening of extraction time, thus significantly higher TPC, TFC, and TNFC were achieved in only 5 min during ultrasound treatment in probe system compared to the half an hour treatment in the ultrasonic bath. In extraction with 50% ethanol even 13% higher TPC was recorded in the sample treated for 5 min in ultrasonic probe system compared to the 30 min in the ultrasonic bath, while in extraction with 80% ethanol those differences were even more pronounced, even 3 times higher TPC was recorded in the sample treated for 5 min in ultrasonic probe system compared to the 30 min in the ultrasonic bath. Also, mild temperature increase during UAE (Figures 1–4) further affected the enhancement of polyphenol solubility thus providing higher polyphenol yields in the UAE-treated extracts, with an emphasis on those samples treated in ultrasonic probe system with the highest TPC. Since the type of solvent and its polarity are one of the factors that strongly influence the yield of the extracted compounds, these properties should also be optimized and carefully selected for each chemical compound to be extracted. However, besides the solvent properties, which are primarily optimized to the solubility of each chemical component, some other properties should also be taken into account, especially the impact on the environment. So in this case, it is preferable to use environmental friendly solvents. The list of solvents that can be used for the extraction of compounds from food and food ingredients is regulated by the European Directive 2010/59/EU (65), according to which ethanol is the preferred solvent due to its lower toxicity. So far, still the most used solvent for the extraction of polar polyphenolic compounds is ethanol, and mixtures of ethanol with water at different proportions (40). The hydroethanolic solution in the extraction of polyphenolic compounds also depends on the food matrix or the type of plant material from which the phenols are isolated. In this study, for UAE extraction of TPC, TFC, and TNFC from nettle, the use of a lower ethanol concentration (50% v/v) significantly contributed to obtain higher TPC yields, as much as 68% higher than the 80% hydroethanolic solution, regardless of the type of ultrasonic equipment used. The same trend of hydroethanolic concentration on polyphenolic compounds was also observed in the nettle samples prepared by classical extraction. As mentioned above, the efficiency of the hydroethanolic solution also depends on the food matrix. For example, authors Bamba et al. (66) found a higher efficiency of 50% ethanol (v/v) in the extraction of TFC from blueberry pomace, while authors Aourabi et al. (67) indicated a higher yield of TFC from corn waste when 70% ethanol (v/v) was used. Another phenomenon caused by the process of sonolysis during UAE is increase in the degree of hydroxylation of polyphenolic compounds induced by the formation of OH- radicals as a direct result of sonolysis process on the water molecules in the matrix, thus generally affecting the improvement of the functionality and bioavailability of polyphenols (68, 69). Of course, other important variable in terms of the efficiency of polyphenol extraction besides the extraction method is also solvent type.

### Pigment Compounds Content of Nettle Alcoholic Extracts

Chlorophylls and carotenoids as the main pigments in nettle leaves play a crucial role in the life of plant organisms, i.e., in photosynthesis, and have a significant functional role in human organisms associated with human health benefits. Chlorophylls are potent chelating agents that have strong antioxidant and anti-inflammatory effects, tend to repair cells, and increase hemoglobin levels in the blood, while both, together with carotenoids, have antimutagenic and anticarcinogenic effects and antiseptic activity. In addition to their pharmaceutical potential, both have been used in the food and cosmetic industries as valuable natural pigment ingredients in various foods and cosmetics (70, 71). Since the preservation of pigmented compounds is very challenging due to the extreme sensitivity to process conditions such as system temperature, process duration, exposure to light, and oxidation tendency, it is necessary to combine the less invasive techniques, such as UAE. As the results of this study also show (Table 3), UAE contributes positively to the preservation of pigment compounds (total chlorophylls and carotenoids), but with the necessary optimization of key factors such as equipment, duration, and the solvent used. In general, the highest total chlorophyll content (TCh, 72.23 μg/g) and total carotenoid content (TCa, 16.75 μg/g) were achieved when treated with the probe system for 15 min and using 80% v/v ethanol as solvent. First, it should be highlighted that both the device and the solvent type used (EM × S) had the greatest influence on TCh and TCa (*p* ≤ 0.0001). When considering solvent type, significantly higher pigment values were obtained when 80% v/v ethanol was used, regardless of extraction method and duration, which is consistent with other literature data that also suggest 80% v/v ethanol as an optimal solvent for pigment isolation (42, 70). Furthermore, if we compare the efficiency of each of the devices used for UAE, we can see that the probe system was significantly more efficient even when using 50 and 80% ethanol for both TCh and TCa. For extraction with 50% v/v ethanol, as much as 63% higher TCh and about 9% higher TCa content were observed with the probe system compared to the ultrasonic bath. For extraction with 80% v/v ethanol this trend was even more pronounced, with about 6 times higher TCh and about 47% higher TCa content using the probe system compared to extraction in the bath. From all of this can be concluded that UAE is an efficient method of pigment extraction with the significant preservation of its content but with the necessary optimization of key factors as suggested from other literature data (70–72).

**Table 3:** Pigment compounds content of nettle alcoholic extracts.

**Sample ID**	**Chl_a** **(μg/g)**	**Chl_b** **(μg/g)**	**TCh** **(μg/g)**	**TCa** **(μg/g)**
Classic extraction				
SL-50	0.41^h^ ± 0.02	0.56^gh^ ± 0.03	0.97^gh^ ± 0.05	1.47^h^ ± 0.01
SL-80	32.96^c^ ± 0.23	15.62^c^ ± 0.09	48.59^c^ ± 0.31	15.30^c^ ± 0.04
Ultrasonic-assisted extraction				
PS-50-5	0.39^hi^ ± 0.01	0.44^h^ ± 0.02	0.83^gh^ ± 0.02	1.51^h^ ± 0.01
PS-50-10	0.49^h^ ± 0.01	0.46^h^ ± 0.01	0.95^gh^ ± 0.01	1.59^h^ ± 0.01
PS-50-15	0.84^g^ ± 0.04	0.71^g^ ± 0.02	1.10^g^ ± 0.82	1.83^g^ ± 0.01
B-50-10	0.24^ij^ ± 0.01	0.38^h^ ± 0.01	0.64^gh^ ± 0.02	1.55^h^ ± 0.01
B-50-15	0.20^j^ ± 0.01	0.38^h^ ± 0.01	0.58^h^ ± 0.01	1.46^h^ ± 0.01
B-50-30	0.21^j^ ± 0.01	0.44^h^ ± 0.17	0.56^h^ ± 0.02	1.49^h^ ± 0.01
PS-80-5	21.99^d^ ± 0.16	10.31^d^ ± 0.08	32.30^d^ ± 0.23	11.32^d^ ± 0.04
PS-80-10	36.59^b^ ± 0.07	18.39^b^ ± 0.30	55.14^b^ ± 0.11	16.28^b^ ± 0.01
PS-80-15	46.61^a^ ± 0.10	26.62^a^ ± 0.13	73.23^a^ ± 0.22	16.75^a^ ± 0.30
B-80-10	6.16^e^ ± 0.04	2.81^e^ ± 0.01	8.97^e^ ± 0.05	3.50^e^ ± 0.01
B-80-15	5.02^f^ ± 0.01	2.32^f^ ± 0.01	7.34^f^ ± 0.01	2.93^f^ ± 0.01
B-80-30	6.13^e^ ± 0.02	2.80^e^ ± 0.04	8.93^e^ ± 0.06	3.65^e^ ± 0.01
ANOVA	*p* ≤ 0.0001	*p* ≤ 0.0001	*p* ≤ 0.0001	*p* ≤ 0.0001
LSD	0.1628	0.2114	0.514	0.186
EM × S	0.0001	0.0001	0.0001	0.0001
EM × T	0.6374	0.4787	0.5925	0.8850
S × T	0.3522	0.4085	0.3782	0.2151
EM × S × T	0.0001	0.0001	0.0001	0.0001

*Chl_a, chlorophyll a content; Chl_b, chlorophyll b content; TCh, total chlorophyll content; TCa, total carotenoid content; EM × S, the interaction of extraction method and solvent concentration; EM × T, the interaction of extraction method and time; S × T, the interaction of solvent concentration and time; EM × S × T, the interaction of extraction method, solvent concentration and time. Results are expressed as mean ± standard deviation. Different letters indicate significant differences between mean values*.

### Antioxidant Capacity of Nettle Alcoholic Extracts

Based on the obtained high content of the specialized metabolites, high values in antioxidant capacity were found for all alcoholic nettle extracts, as expected (Table 2). In addition, a significant effect of all varied variables, especially solvent concentration and time, on the antioxidant capacity of the nettle extracts was found. In general, higher antioxidant capacity values were obtained for nettle extracts prepared with 80% v/v, regardless of the ultrasonic device used and the duration of extraction, except for sample B-80-15, where time (i.e., too short treatment) influenced the lower antioxidant capacity value. When extracted with 50% v/v ethanol, slightly higher antioxidant capacity values were obtained for the samples treated in the ultrasonic bath than for those treated in the ultrasonic probe system. This trend was not observed for the extraction with 80% v/v ethanol, as significantly higher antioxidant capacity values were obtained for the stinging nettle extracts treated in the probe system, about 12% higher values compared to the values obtained for the extraction in the bath. Considering that the results of the analysis of specialized metabolites, showed a significant beneficial effect on UAE, it is expected that these samples can be characterized as having a strong antioxidant capacity. Other studies also indicate a positive effect of ultrasound on the antioxidant properties of various plant matrices (72–74) and can therefore be considered as an efficient means of obtaining plant products with better nutritional and thus biological properties.

### Physicochemical Parameters of Nettle Alcoholic Extracts

The efficiency of extraction assisted by high-intensity ultrasound depends mainly on several variables, some of which are related to the type of solvent or, in particular, to some of the properties, such as viscosity, density, surface tension (medium), and presence of solid particles (physical properties) (41, 42). Density, viscosity, and acoustic impedance are the most important solvent properties that strongly affect extraction efficiency, as they have a major impact on the amount of acoustic power. As noted by authors Kobus and Kusińska (41), the aforementioned variables are critical to the amount of energy required by the ultrasonic processor to maintain a constant amplitude. In general, the power density generated by the ultrasonic processor in water is higher than the power density in ethyl alcohol. In addition to density, other physicochemical properties such as electrical conductivity (EC), organic acid content (TA), and pH of extract provide additional information about the purity, concentration of ingredients, composition and stability, quality, behavior, and final use (75). It should be emphasized that all these parameters are significantly affected by the temperature of the system as a direct result of the cavitation process. According to the results of this study (Table 2), the density of the alcoholic nettle extracts was significantly influenced by the ethanol concentration, while the extraction method (classical and UAE both with the probe system and in the bath) had no significant influence. In general, as expected, the density of the nettle extracts was higher when 50% v/v ethanol was used (average value regardless of extraction method 0.9648 g/cm^3^) compared to 80% v/v (average value regardless of extraction method 0.8598 g/cm^3^) and was not affected by the type of treatment or extraction method. EC values were both affected by ethanol concentration, method, and time of the extraction. First, the extraction method (classic or UAE) significantly affected the EC values of alcoholic nettle extracts, during which significantly higher EC values were observed during the UAE treatment regardless of the type of equipment used and duration of extraction. When using 50% v/v ethanol even 2.5 times higher EC values were determined in UAE-treated samples, while using 80% v/v ethanol about even 8 times higher values were determined compared to the classically extracted samples. Indeed, the electrical conductivity of a solution, an extract, depends primarily on the content of dissolved nutrients such as minerals, vitamins, proteins, etc. Ultrasonic treatment could facilitate the release of these compounds contained in the cells or colloidal particles of the plant material, due to the transient cavitation effects (mechanical damage to the plant tissue). Moreover, during UAE treatment, the temperature of the medium increases (Figures 1–4), which results in the solutes or their ionic forms acquiring higher kinetic energy to overcome the intermolecular forces, leading to their easier movement, i.e., mass transfer into the surrounding solvent (76). TA and pH values (Table 4) also significantly differ considering the varied factors, respectively extraction method, hydroethanolic solution, and time, with in general higher values in UAE-treated nettle extracts prepared both with 50 and 80% v/v ethanol regardless of the ultrasonic system (probe or bath) and time of the extraction. Considering the ethanol concentration, 50% v/v ethanol was more efficient for the extraction of organic acids from plant material, which was expected due to the higher water share and greater solubility of organic acids in 50% v/v compared to the 80% v/v ethanol. Furthermore, regarding the ultrasonic system varied, extraction in ultrasonic bath resulted in higher yields of TA, with the highest determined value of 1.16%, when using 50% v/v ethanol. In extraction with 80% v/v ethanol those results were contrary, with on average higher TA content in samples treated by the ultrasonic probe. pH values as expected for all prepared alcoholic nettle extracts were in a neutral and slightly acidic range and in line with the lower content of TA in all nettle extracts. In general, other literature data also suggest a significant positive impact of sonication treatment on most of the physicochemical parameters of nettle extracts studied, rather on density, electrical conductivity, pH, and total acid content (34, 75, 77, 78).

**Table 4:** Physicochemical parameters of nettle alcoholic extracts.

**Sample ID**	**Density (g/cm^**3**^)**	**EC (μS/cm)**	**TA (%)**	**pH**
Classic extraction				
SL-50	0.9664^a^ ± 0.01	285^bcd^ ± 0.06	0.2^f^ ± 0.01	7.8^a^ ± 0.11
SL-80	0.8611^b^ ± 0.01	24.27^d^ ± 1.00	0.36^cde^ ± 0.05	7.11^bcd^ ± 0.24
Ultrasonic-assisted extraction				
PS-50-5	0.9681^a^ ± 0.01	874^a^ ± 2.65	0.6^b^ ± 0.16	6.48^efg^ ± 0.35
PS-50-10	0.9531^a^ ± 0.03	1,000^a^ ± 1.00	0.44^bcde^ ± 0.03	7.21^b^ ± 0.06
PS-50-15	0.9721^a^ ± 0.01	304^bc^ ± 1.00	0.43^bcde^ ± 0.03	7.18^b^ ± 0.02
B-50-10	0.9673^a^ ± 0.01	902^a^ ± 1.00	0.43^bcde^ ± 0.03	6.72^def^ ± 0.17
B-50-15	0.9584^a^ ± 0.01	876^a^ ± 1.00	1.16^a^ ± 0.16	6.97 ^bcd^ ± 0.30
B-50-30	0.9685^a^ ± 0.01	944^a^ ± 1.00	0.49^bc^ ± 0.10	7.15 ^bc^ ± 0.16
PS-80-5	0.8613^b^ ± 0.01	260.67^bcd^ ± 0.58	0.44^bcde^ ± 0.08	6.19^g^ ± 0.14
PS-80-10	0.8615^b^ ± 0.01	304^bc^ ± 1.00	0.49^bc^ ± 0.15	6.21 ^g^ ± 0.17
PS-80-15	0.8543^b^ ± 0.02	325^bc^ ± 1.00	0.55^bc^ ± 0.11	6.43^efg^ ± 0.06
B-80-10	0.8609^b^ ± 0.01	332^b^ ± 0.10	0.29^de^ ± 0.03	6.78 ^cde^ ± 0.05
B-80-15	0.8599^b^ ± 0.01	69.93^cd^ ± 0.35	0.25^e^ ± 0.03	6.38^fg^ ± 0.10
B-80-30	0.8601^b^ ± 0.01	87.43^cd^ ± 0.15	0.26^e^ ± 0.02	6.50^efg^ ± 0.16
ANOVA	*p* ≤ 0.0001	*p* ≤ 0.0001	*p* ≤ 0.0001	*p* ≤ s0.0001
LSD	0.0209	261.25	0.1957	0.3924
EM × S	0.9885	0.0001	0.0002	0.2143
EM × T	0.8241	0.5101	0.0959	0.5982
S × T	0.7856	0.0001	0.0131	0.4573
EM × S × T	0.2731	0.0001	0.0001	0.0005

*EC, electrical conductivity; TA, total acid content; EM × S, the interaction of extraction method and solvent concentration; EM × T, the interaction of extraction method and time; S × T, the interaction of solvent concentration and time; EM × S × T, the interaction of extraction method, solvent concentration and time. Results are expressed as mean ± standard deviation. Different letters indicate significant differences between mean values*.

**Figure 5:**
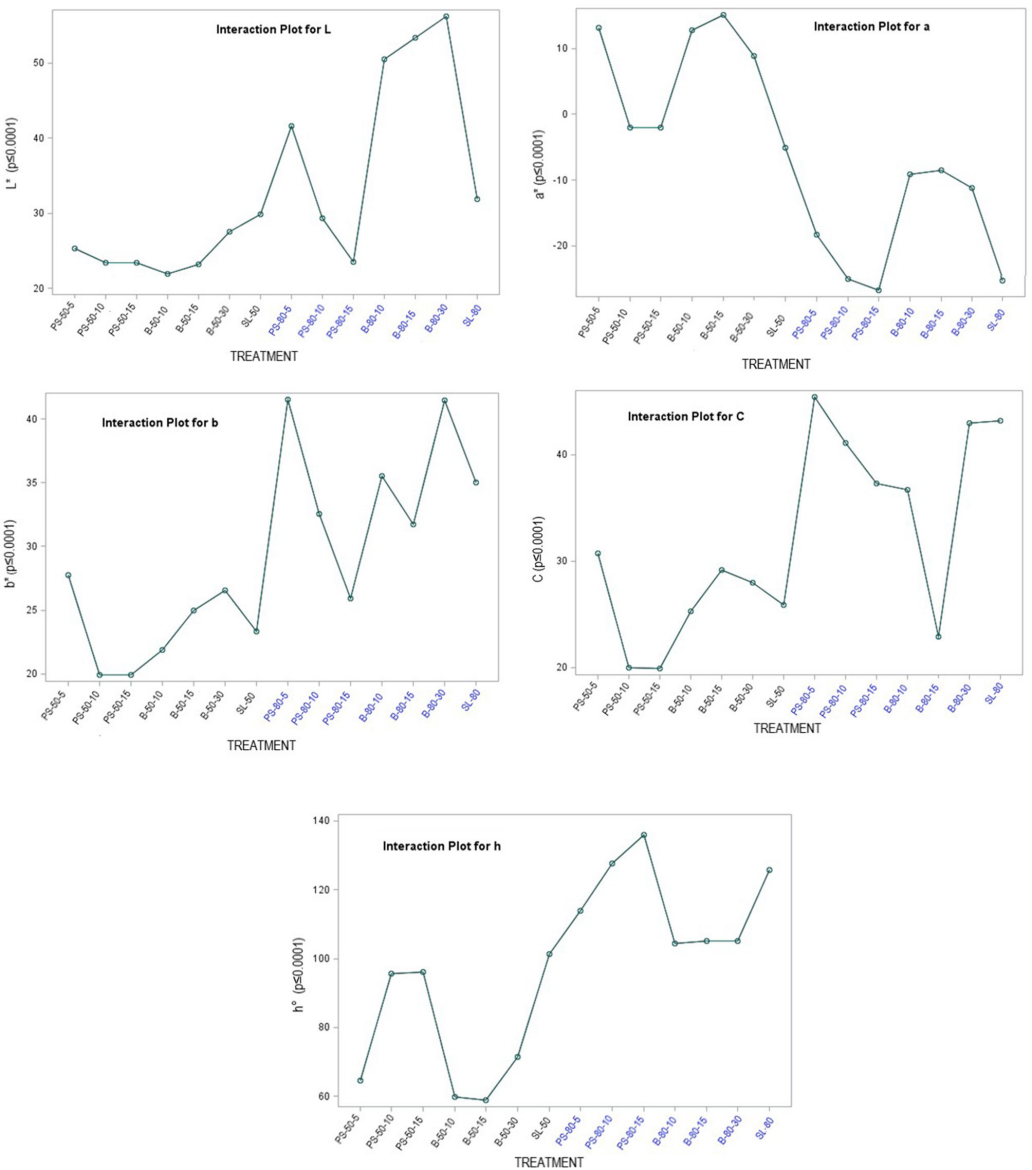
Chromaticity parameters of nettle alcoholic extracts: L* value; a* value; b* value; C value; h° value.

The color of a product is often associated by consumers with its quality and is therefore considered as one of the most important external parameters. Indeed, food processing techniques primarily affect the external characteristics of a product, and optimization of their parameters is necessary to maintain the color of the final product. The increased temperature during processing is one of the factors that significantly affect color, mainly by accelerating enzymatic and metabolic processes in the plant material, which also translates into color changes. Non-invasive techniques such as ultrasound are therefore very effective in maintaining external quality parameters, as they do not cause a critical increase in system temperature and significantly reduce time, but optimization of process parameters is necessary to avoid side effects (79–81). The chromaticity parameters of the alcoholic nettle extracts (L^*^, a^*^, b^*^, C, h°) varied significantly (*p* ≤ 0.0001) depending on different factors combined (Figure 5). The analysis of the significance of the interactions of the different factors (Table 5) showed that, besides the combination of all three different factors (EM × S × T), the interaction between the extraction method and the solvent type (EM × S) had a significant effect on the values of the chromaticity parameters, L^*^ and C. As can be seen from the interaction plot (Figure 5), L^*^ values were generally lower when extracted with 50% v/v ethanol regardless of the extraction method, indicating a darker color of the extracts. Regardless of the extraction method, alcoholic nettle extracts prepared with 80% v/v ethanol had significantly lower (-) a^*^ values, indicating a greener color of these extracts. As for the ultrasonic device used, lower values of all analyzed color parameters were obtained on average during the ultrasonic probe treatment, proving the preservation of the specific green color, which is consistent with the higher values of total chlorophylls during the treatment with 80% v/v ethanol using the ultrasonic probe. To investigate the relationship between color values and pigment content, a scatter plot (not shown) and regression analysis (the first and second-order modeling) were performed between each color parameter (L^*^, a^*^, b^*^, C, h°) and chlorophyll a, b, total chlorophyll, and total carotenoid content. The best fit is shown in Table 6. In the case of luminosity, the scatter plot shows two populations, a very scattered one corresponding to the extracts obtained with 50% ethanol and another population with high linearity corresponding to the extracts obtained with 80% ethanol. Consequently, the luminosity model given in Table 6 allows the prediction of the concentration of chlorophyll a, b, total chlorophyll, and total carotenoids based on the luminosity value of the nettle extracts obtained with 80% ethanol as solvent. As can be observed, there is an inversely proportional relationship between the parameters, i.e., as the concentration of chlorophylls and carotenoids increases, the luminosity value decreases. For a^*^, the second-order model was found to best fit the values of a^*^ and the concentration of pigments. In this case, there is a direct correlation, as the concentration of pigments increases, the value of a^*^ increases. The last model obtained relates the hue (h°) to the concentration of pigments. Also in this case, the second-order equation is the one that best fits the data. For b^*^ and chroma, it was not possible to obtain models with a high *R*^2^ that could be used to describe the chlorophyll and carotenoid content.

**Table 5:** Significance of interactions of varied factors in the treatment of nettle alcoholic extracts for chromaticity parameters.

**Interactions**	L*	a*	b*	**C**	**h**°****
EM × S	0.0001	0.1751	0.9404	0.0088	0.1456
EM × T	0.5916	0.8296	0.5851	0.6667	0.8033
S × T	0.1071	0.6383	0.0002	0.0161	0.5963
EM × S × T	0.0001	0.0001	0.0001	0.0001	0.0001

*EM × S, the interaction of extraction method and solvent concentration; EM × T, the interaction of extraction method and time; S × T, the interaction of solvent concentration and time; EM × S × T, the interaction of extraction method, solvent concentration and time. Results are expressed as mean ± standard deviation. Different letters indicate significant differences between mean values*.

**Table 6:** Regression results for color values vs. pigment content.

	**Chl_a**	**Chl_b**	**TCh**	**TCa**
L	y = −1.30x + 75.50 *R*^2^ = 0.980	y = −0.71x + 40.38 *R*^2^ = 0.956	y = −2.02x + 115.98 *R*^2^ = 0.975	y = −0.49x + 29.79 *R*^2^ = 0.949
a*	y = 0.05x^2^ – 0.43x – 1.69 *R*^2^ = 0.981	y = 0.02x^2^ – 0.20x – 0.98 *R*^2^ = 0.949	y = 0.07x^2^ – 0.62x – 2.75 *R*^2^ = 0.973	y = 0.02x^2^ – 0.17x + 0.83 *R*^2^ = 0.984
h°	y = 0.02x^2^ – 2.50x + 93.79 *R*^2^ = 0.974	y = 0.01x^2^ – 1.41x + 53.83 *R*^2^ = 0.981	y = 0.03x^2^ – 3.93x + 148.20 *R*^2^ = 0.979	y = 0.01x^2^ – 0.88x + 33.96 *R*^2^ = 0.923

Chl_a, chlorophyll a content; Chl_b, chlorophyll b content; TCh, total chlorophyll content; TCa, total carotenoid content. In the equations, the “x” corresponds to L, a*, and h°, respectively (by rows) and the “y” corresponds to Chl_a, Chl_b, TCh and TCa, respectively (by column). R^2^ is the coefficient of determination.

### Principal Component Analysis Results

**Figure 6:**
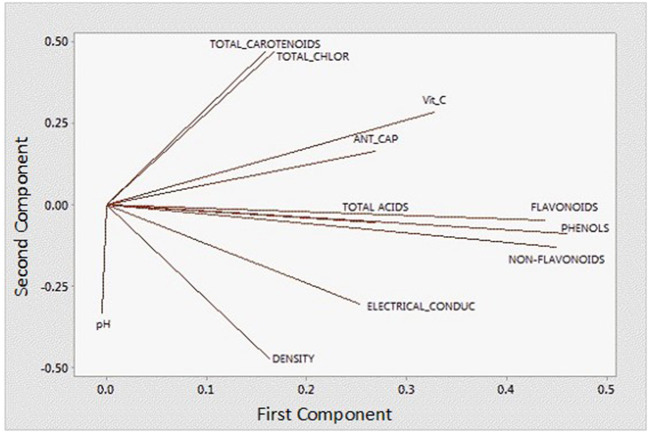
Loading plot for the variables investigated by principal component analysis.

**Figure 7:**
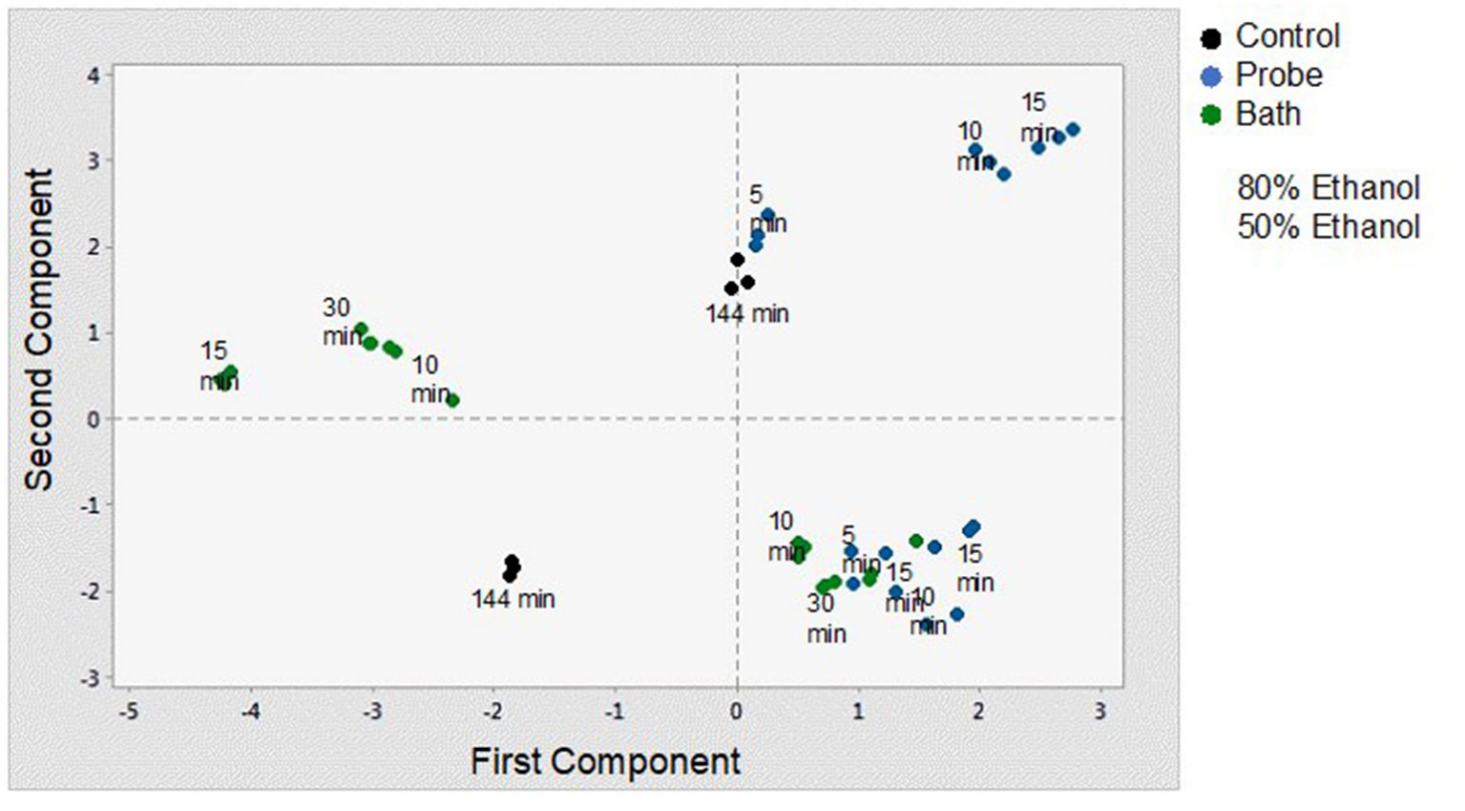
Score plot for the principal components analysis.

In order to better understand and visualize the data obtained, a PCA analysis was performed. The first principal component (PC) is responsible for 38.7% of the total variance. The variables most correlated with the first principal component (PC1) are phenolic content (0.449), non-flavonoids (0.449), flavonoids (0.438), vitamin C content (0.328), and antioxidant capacity (0.269). The second PC accounts for 33.3% of the total variance and correlates most strongly with density (-0.471), total chlorophyll (0.466), total carotenoids (0.469), and pH (−0.333). Thus, these first two principal components explain 72% of the variation in the data. From Figure 6 and the correlation coefficients, it can be deduced that the first PC primarily measures extraction from the point of view of compounds with antioxidant capacity and the second PC considers the influence of ethanol concentration on the properties of the extract. In Figure 7, you can see how the data are grouped according to the first two components. Five groups can be distinguished. For the samples extracted with 50% ethanol, there are two groups, one containing the control samples and the other grouping all samples extracted with 50% ethanol, regardless of the type of US used. For the samples extracted with 80% ethanol, three groups can be distinguished. One group includes the samples extracted by 5, 10, or 30 min of US treatment in a bath; another group includes the samples extracted by 5 min of US treatment with a probe and the control sample; and finally, a group that includes the samples extracted by 10 and 15 min of US probe.

## Data Availability Statement

The raw data supporting the conclusions of this article will be made available by the authors, without undue reservation.

## Author Contributions

JŠŽ: conceptualization and writing–original draft preparation. JŠŽ, SR, MB, and FB: methodology. FB and JC: software. AR, MD, SV, and JC: data curation. MB, FB, and SV: writing–review and editing. MD: visualization. All authors have read and agreed to the published version of the manuscript.

## Funding

This research was funded by Croatian Science Foundation (Hrvatska zaklada za znanost–www.hrzz.hr) under the project IP-2019-04-3325 URTICA-BioFuture–Nutritional and functional value of nettle (*Urtica dioica* L.) by application of modern hydroponic cultivation techniques. Juan Manuel Castagnini thanks the University of Valencia for the Maria Zambrano postdoctoral contract (ZA21-028) through the project Extraction of bioactive compounds from food matrices using innovative and sustainable technologies (EXTRABIO).

## Conflict of Interest

The authors declare that the research was conducted in the absence of any commercial or financial relationships that could be construed as a potential conflict of interest.

## Publisher's Note

All claims expressed in this article are solely those of the authors and do not necessarily represent those of their affiliated organizations, or those of the publisher, the editors and the reviewers. Any product that may be evaluated in this article, or claim that may be made by its manufacturer, is not guaranteed or endorsed by the publisher.
